# Complementary feeding introduction methods and pediatric feeding disorder in the first year of life: a randomized clinical trial

**DOI:** 10.1590/2317-1782/e20250265en

**Published:** 2026-07-16

**Authors:** Letícia Cristine Welke, Adriela Azevedo Souza Mariath, Juliana Rombaldi Bernardi, Leandro Meirelles Nunes, Erissandra Gomes

**Affiliations:** 1 Curso de Graduação em Fonoaudiologia, Faculdade de Odontologia, Universidade Federal do Rio Grande do Sul – UFRGS - Porto Alegre (RS), Brasil.; 2 Departamento de Cirurgia e Ortopedia, Faculdade de Odontologia, Universidade Federal do Rio Grande do Sul – UFRGS - Porto Alegre (RS), Brasil.; 3 Departamento de Nutrição, Faculdade de Medicina, Universidade Federal do Rio Grande do Sul – UFRGS - Porto Alegre (RS), Brasil.; 4 Hospital de Clínicas de Porto Alegre - Porto Alegre (RS), Brasil.; 5 Departamento de Pediatria, Faculdade de Medicina, Universidade Federal do Rio Grande do Sul – UFRGS - Porto Alegre (RS), Brasil.

**Keywords:** Feeding Behavior, Infant, Child Feeding, Complementary Feeding, Feeding Methods

## Abstract

**Purpose:**

To investigate whether the method of introducing complementary feeding (CF) is related to pediatric feeding disorders (PFD) in the first year of life.

**Methods:**

Clinical trial with 128 mother-child pairs randomized into intervention groups by CF methods: traditional, Baby-Led Introduction to Solids (BLISS), or mixed. The intervention was carried out at the infants’ 5 and a half months of age in an experimental kitchen with meals prepared according to the randomized method. Follow-up was conducted at their 7 and 9 months of age. At 12 months, the Brazilian Child Feeding Scale (EBAI) was applied to screen for PFD. This study was registered in the Brazilian Clinical Trials Registry (ReBEC).

**Results:**

PFD was present in 16 (12.5%) children: 5 (3.9%) from the traditional method (3 with mild PFD and 2 with severe PFD), 9 (7%) from the BLISS method (5 with mild PFD, 2 with moderate PFD, and 2 with severe PFD), and 2 (1.6%) from the mixed method (both with mild PFD). In the analysis of the 14 EBAI questions, the responses tended towards the adequate and/or favorable aspects across all CF methods, with no statistically significant differences.

**Conclusion:**

PFD was not influenced by the CF method in the study sample.

## INTRODUCTION

Complementary feeding (CF) to breast milk or infant formula is introduced by presenting other liquids and, especially, solid foods. For CF to be introduced , infants must show signs of readiness and skills associated with cervical and gross and fine motor control, development of oral aspects, demonstration of interest in food, and gastrointestinal and immune system maturation^(1,2)^.

The Brazilian Ministry of Health recommends that CF be introduced at 6 months of age, with the child's intake being monitored by the caregiver, initially offering foods in the texture of porridges and/or purees, using utensils, and gradually approaching the consistency of family food at 1 year of age^(1)^. This method has been described as traditional or Parent-Led Weaning (PLW). However, in recent years, the literature has considered other CF methods that promote the child's autonomy^(3-5)^. Baby-Led Weaning (BLW) is a CF introduction method that allows the child to guide the feeding time, with the main objective of developing the notion of satiety and autonomy, offering foods in the form of strips or sticks, and not adapting their consistencies^(6)^. Considering the questions raised by researchers and health professionals regarding the BLW method, a modified version was created, called Baby-Led Introduction to Solids (BLISS). The BLISS recommendations are the same as the BLW method, but with some additional aspects: ensuring a source of iron-rich foods at each meal, offering a high-calorie food at each meal, offering foods prepared in a texture and shape that reduces the risk of choking, and avoiding foods listed as high risk of aspiration^(7)^.

During the infant's CF introduction process, feeding difficulties may occur, which, if not observed and properly addressed, can result in pediatric feeding disorder (PFD) and persist throughout childhood development. PFD is defined as impaired or inappropriate oral intake for age, associated with a medical, nutritional, dietary, and/or psychosocial condition^(8)^. PFD can occur in children with neurotypical development, with a prevalence of 25-35%, as well as in up to 80% of those with comorbidities. Clinical manifestations range from atypical behaviors to altered clinical aspects, affecting overall development and biopsychosocial issues, especially family relationships and quality of life^(9,10)^. Although there are different protocols for screening PFD in the literature, one of the most used and the only one validated in Brazil is the Montreal Children’s Hospital Feeding Scale (MCH-FS), nationally known as the Brazilian Child Feeding Scale (EBAI)^(11)^.

CF is a major concern for parents and guardians of infants, especially when feeding difficulties occur in the food introduction process, which can culminate in PFD and cause various consequences for the child^(12)^. Considering that CF is an important moment in child development and for a proper relationship between the child and food, this study is justified by the importance of research focused on the relationship between CF methods and PFD. Thus, this study aimed to verify whether the CF introduction method is related to PFD in the first year of an infant's life.

## METHODS

This research is part of a larger study that was approved by the Research Ethics Committee of the Clinics Hospital of Porto Alegre, Brazil, under approval number 3.094.555 (CAAE: 01537018.5.0000.5327) and registered in the Brazilian Clinical Trials Registry (ReBEC) with number RBR-229SCM. The children's mothers agreed to participate in the study by signing an informed consent form. The protocol of the study to which this belongs has been previously published^(5)^. This is a randomized controlled clinical trial with three intervention arms regarding the CF introduction method:

- traditional method^(1)^: Parents were instructed to start offering CF slowly and gradually by spoon from the 6^th^ month of life; to offer CF (cereals, tubers, meats, legumes, fruits, and vegetables) three times a day, without rigid schedules and respecting appetite, increasing the offer over the months; the food consistency should initially be pasty, mashed with a fork (from 6 to 8 months in the form of porridge and puree) and gradually progress until it reaches the consistency of the family's food at 12 months of age, with a variety of colors and food groups in all meals, without mixing or straining the food.
- BLISS method^(5)^: Parents were instructed to encourage the infant to feed themselves, but always accompanied by an adult and participating in family meals. The consistency of the food offered from 6 months of age should be firm, in shapes that allow the infant to feed themselves with their own hands; cut into elongated shapes, such as strips or sticks, to facilitate the pincer movement of the fingers and avoid choking. They were advised to respect the child's time to explore flavors and textures, and to offer three types of food at each meal: a food source of iron (e.g., red meat), a food source of energy, and a food source of fiber (e.g., fruits and vegetables).
- Mixed method^(1)^: Parents were instructed to initially apply the BLISS method. If the child was not satisfied or uninterested in the food, they were instructed to offer the food using the traditional method during the same meal. Caregivers were trained and received printed material on how to perceive the child's signs of satisfaction. This approach considers the child's desire and behavior.

The sample consisted of mothers residing in Porto Alegre and the metropolitan region and their healthy, full-term, non-twin children, weighing 2,500 grams or more at birth, who had not yet started CF. Exclusion criteria were children with congenital malformations, neurological deficits, or dietary restrictions in the first year of life. They were recruited through online invitations on social media, webpages, and groups related to mothers and through posters in locations relevant to the research topic. A researcher not involved in data collection or analysis randomized the sample in blocks of three until reaching the calculated number of pairs, using the Randomization website^(13)^.

After group randomization, intervention meetings were held with groups of four to eight mothers when the infants reached 5 and a half months of age, at a nutrition clinic, with a nutritionist and a speech-language-hearing pathologist. Due to the COVID-19 pandemic, some interventions took place remotely, via video call on virtual communication applications, with the same guidelines and team involved in the in-person intervention. Guidance on infant feeding was reinforced through telephone contact at 7 months and home visits at 9 months. They were assessed at 12 months of age in an in-person meeting or remotely. This study collected data from an initial questionnaire to characterize the sample and at 12 months to apply EBAI and obtain general, dietary, and oral health data. Both were applied remotely and self-answered by parents individually. Then, they sent them to the researchers, who were available to answer any questions about completing it.

The EBAI aims to assess children’s feeding behavior. The original MCH-FS protocol was published in Canada in 2011 to classify difficulties based on the scores; a T-score between 61 and 65 is classified as mild difficulty, 66 to 70 as moderate difficulty, and above 70 as severe difficulty. It is characterized as a simple and quick screening tool for feeding problems in children aged 6 months to 6 years and 11 months. It has 14 items to be answered by the caregiver who is present at most of the child's meals, with answers ranging from 1 to 7, with a classification for each variable. The items are related to oral sensorimotor symptoms, oral sensory symptoms, appetite, concerns and behaviors at mealtimes, strategies used, and caregiver reactions^(14)^. The MCH-FS was validated in 2011 in Canada, its country of origin, and in other countries; in Brazil, it was validated and adapted for Brazilian Portuguese, resulting in the EBAI^(11)^.

The database was created using the Statistical Package for the Social Sciences (SPSS), version 21.0, with double entry and subsequent validation. Each mother-baby pair was identified with a number to maintain anonymity. Categorical variables were presented as relative and absolute percentages, and quantitative variables as mean (standard deviation), median (minimum and maximum), or median (Q_1_ and Q_3_). The study used a significance level of p < 5%, employing Pearson's chi-square test and ANOVA test.

## RESULTS

**Figure 1:**
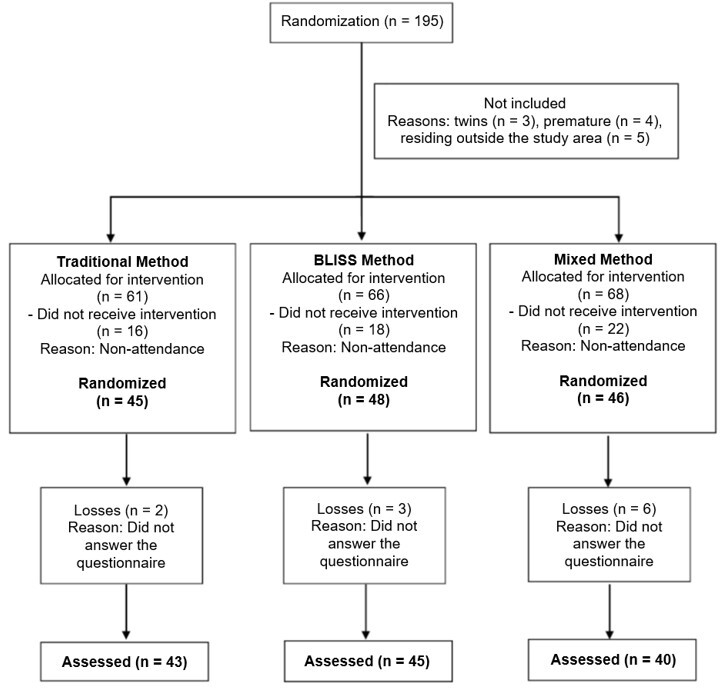
Study flowchart

The sample consisted of 128 mother-infant pairs, divided by CF method: 43 (33.6%) in the traditional method, 45 (35.1%) in the BLISS method, and 40 (31.2%) in the mixed method, as shown in the flowchart in Figure 1. The initial characterization data of the total sample and by the CF method are described in Table 1, and the data at 12 months are shown in Table 2. The variables described with the CF methods were not significantly different, except for “attends daycare” (p < 0.05), though not affecting the final comparative analysis.

Caption: BLISS = Baby-Led Introduction to Solids

**Table 1:** Characterization of the total sample and by the CF introduction method at 5 and a half months of age (n = 128)

**Variables**	**Total**	**Methods**			**p-value**
		**Traditional n = 43**	**BLISS n = 45**	**Mixed n = 40**	
**Child’s sex** – n(%)					
Males	58(45.3)	17(39.5)	21(46.7)	20(50)	0.617^*^
Females	70(54.7)	26(60.5)	24(53.3)	20(50)	
**Mother’s education** – mean±standard deviation					
	17.6±5.3	16.9±5.3	18±6.1	17.9±4.4	0.627^**^
**Mother’s race** – n(%)					0.697*
White	109(85.2)	35(81.4)	38(84.4)	36(90)	
Others	19(14.8)	8(18.6)	7(15.6)	4(10)	
**Family income (R$)** –					
Median	6000	5000	8000	6000	0.164**
(Q_1_-Q_3_)	(4000 - 10000)	(3700 - 10000)	(4000 - 14500)	(4550 - 10000)	

* Pearson's chi-square test;

** ANOVA test; p < 0.05

Caption: BLISS = Baby-Led Introduction to Solids; CF = complementary feeding

**Table 2:** Characterization of the total sample and by the CF introduction method at 12 months (n = 128)

**Variables**	**Total**	**Methods**			
		**Traditional** **n = 43**	**BLISS** **n = 45**	**Mixed** **n = 40**	**p-value**
**Child’s weight (kg)** – mean±standard deviation					
	9.6±1.2	9.8±1.4	9.5±1.0	9.6±1.1	0.582**
					
**Age at CF introduction (in days)** – mean±standard deviation	172.5±24.2	168.2±28.7	174.0±22.7	175.5±20.1	0.343^**^
**Attends daycare** – n(%)	37(28.9)	18(41.9)	12(26.7)	7(17.5)	0.046^*^

						
**Currently breastfeeding –** n(%)	96(75)	34(79)	34(75.5)	28(70)	0.631*	
**Using baby bottle** – n(%)	77(60.2)	27(62.8)	30(66.7)	20(50)	0.267*	
**Using pacifier** – n(%)						
	42(32.8)	14(32.5)	18(40)	10(25)	0.339*	

* Pearson's chi-square test;

** ANOVA test; p < 0.05

Caption: BLISS = Baby-Led Introduction to Solids; CF = complementary feeding

The EBAI score is described in Table 3. There was no statistically significant difference (p > 0.05) between CF methods. The T-score cutoff for PFD showed that it was present in 16 (12.5%) children: five (3.9%) belonged to the traditional method (three with mild PFD and two with severe PFD), nine (7%) to the BLISS method (five with mild PFD, two with moderate PFD, and two with severe PFD), and two (1.6%) to the mixed method (both classified as mild).

**Table 3:** EBAI by total sample and by the CF introduction method at 12 months of age (n = 128)

**Variables**	**Total**	**Methods**			
		**Traditional n = 43**	**BLISS n = 45**	**Mixed** **n = 40**	**p-value** ^*^
Score					
mean±standard deviation	51.4±7.6	51.2±8.3	52.5±8.5	50.3±5.5	0.394
					
95% CI	50.1-52.7	48.6-53.7	50-55.1	48.6-52.1	
					
median (minimum-maximum)	50 (36-74)	50 (36-74)	51 (40-72)	49 (39-64)	

* ANOVA test; p < 0.05

Caption: BLISS = Baby-Led Introduction to Solids; EBAI = Brazilian Child Feeding Scale; CI = confidence interval; CF = complementary feeding

Chart 1 describes the 14 variables, considering the responses to each of the questions. The analysis of the responses shows that most of the sample attributes favorable characteristics to the CF process, regardless of the group to which they belong. No significant differences (p > 0.05) were found between CF methods regarding the questions.

**Chart 1:** Absolute and relative distribution of responses to the EBAI (n = 128)

**Variables**	**Responses**						
	**1**	**2**	**3**	**4**	**5**	**6**	**7**
	**n (%)**	**n (%)**	**n (%)**	**n (%)**	**n (%)**	**n (%)**	**n (%)**
**1. What do you think about mealtimes with the child?**	**Very difficult**						**Easy**
Total	-	2 (1.6)	13 (10.2)	22 (17.2)	27 (21.1)	34 (26.6)	30 (23.4)
Traditional	-	2 (1.6)	1 (0.8)	8 (6.3)	11 (8.6)	11 (8.6)	10 (7.8)
BLISS	-	-	10 (7.8)	10 (7.8)	6 (4.7)	8 (6.3)	11 (8.6)
Mixed	-	-	2 (1.6)	4 (3.1)	10 (7.8)	15 (11.7)	9 (7)
**2. How concerned are you about your child's feeding?**	**Not concerned**						**Very concerned**
Total	10 (7.8)	10 (7.8)	9 (7)	14 (10.9)	26 (20.3)	23 (18)	36 (28.1)
Traditional	4 (3.1)	3 (2.3)	2 (1.6)	7 (5.5)	8 (6.3)	6 (4.7)	13 (10.2)
BLISS	3 (2.3)	4 (3.1)	4 (3.1)	3 (2.3)	8 (6.3)	9 (7)	14 (10.9)
Mixed	3 (2.3)	3 (2.3)	3 (2.3)	4 (3.1)	10 (7.8)	8 (6.3)	9 (7)
**3. How hungry is your child? How much appetite does he/she have?**	**He/she is never hungry**						**He/she has a great appetite**
Total	-	4 (3.1)	6 (4.7)	17 (13.3)	24 (18.8)	29 (22.7)	48 (37.5)
Traditional	-	2 (1.6)	3 (2.3)	3 (2.3)	6 (4.7)	10 (7.8)	19 (14.8)
BLISS	-	1 (0.8)	2 (1.6)	9 (7)	9 (7)	7 (5.5)	17 (13.3)
Mixed	-	1 (0.8)	1 (0.8)	5 (3.9)	9 (7)	12 (9.4)	12 (9.4)
**4. When does your child start refusing to eat during meals?**	**In the beginning**						**At the end**
Total	6 (4.7)	5 (3.9)	7 (5.5)	19 (14.8)	20 (15.6)	29 (22.7)	42 (32.8)
Traditional	3 (2.3)	2 (1.6)	1 (0.8)	7 (5.5)	4 (3.1)	10 (7.8)	16 (12.5)
BLISS	1 (0.8)	2 (1.6)	4 (3.1)	10 (7.8)	8 (6.3)	7 (5.5)	13 (10.2)
Mixed	2 (1.6)	1 (0.8)	2 (1.6)	2 (1.6)	8 (6.3)	12 (9.4)	13 (10.2)
**5. How long (in minutes) does your child's meal last?**	**01-10**	**11-20**	**21-30**	**31-40**	**41-50**	**51-60**	**> 60**
Total	17 (13.3)	43 (33.6)	42 (32.8)	16 (12.5)	6 (4.7)	2 (1.6)	2 (1.6)
Traditional	12 (9.4)	11 (8.6)	9 (7)	7 (5.5)	2 (1.6)	1 (0.8)	1 (0.8)
BLISS	2 (1.6)	16 (12.5)	19 (14.8)	4 (3.1)	3 (2.3)	1 (0.8)	-
Mixed	3 (2.3)	16 (12.5)	14 (10.9)	5 (3.9)	1 (0.8)	-	1 (0.8)
**6. How does your child behave during mealtimes?**	**Very well**						**Messy, fussy, and whiny**
							5 (3.9)
Total	21 (16.4)	35 (27.3)	19 (14.8)	25 (19.5)	18 (14.1)	5 (3.9)	1 (0.8)
Traditional	10 (7.8)	8 (6.3)	5 (3.9)	11 (8.6)	5 (3.9)	3 (2.3)	4 (3.1)
BLISS	5 (3.9)	13 (10.2)	8 (6.3)	7 (5.5)	7 (5.5)	1 (0.8)	-
Mixed	6 (4.7)	14 (10.9)	6 (4.7)	7 (5.5)	6 (4.7)	1 (0.8)	
**7. Does your child get nauseous, spit up, or vomit after eating certain foods?**	**Never**						**Most of the time**
Total	41 (32)	53 (41.4)	13 (10.2)	9 (7)	8 (6.3)	2 (1.6)	2 (1.6)
Traditional	12 (9.4)	18 (14.1)	5 (3.9)	3 (2.3)	2 (1.6)	2 (1.6)	1 (0.8)
BLISS	11 (8.6)	20 (15.6)	6 (4.7)	3 (2.3)	4 (3.1)	-	1 (0.8)
Mixed	18 (14.1)	15 (11.7)	2 (1.6)	3 (2.3)	2 (1.6)	-	-
							
**8. Does your child keep food stuck in their mouth?**	**Most of the time**						**Never**
Total	2 (1.6)	1 (0.8)	10 (7.8)	5 (3.9)	10 (7.8)	39 (30.5)	61 (47.7)
Traditional	-	-	4 (3.1)	2 (1.6)	5 (3.9)	13 (10.2)	19 (14.8)
BLISS	1 (0.8)	1 (0.8)	2 (1.6)	3 (2.3)	3 (2.3)	12 (9.4)	23 (18)
Mixed	1 (0.8)	-	4 (3.1)	-	2 (1.6)	14 (10.9)	19 (14.8)
**9. Do you need to chase after your child or use distractions (such as toys, TV) during mealtimes to get them to eat?**	**Never**						**Most of the time**
Total	62 (48.4)	26 (20.3)	15 (11.7)	6 (4.7)	10 (7.8)	6 (4.7)	3 (2.3)
Traditional	24 (18.8)	6 (4.7)	4 (3.1)	1 (0.8)	5 (3.9)	1 (0.8)	2 (1.6)
BLISS	21 (16.4)	10 (7.8)	4 (3.1)	2 (1.6)	3 (2.3)	4 (3.1)	1 (0.8)
Mixed	17 (13.3)	10 (7.8)	7 (5.5)	3 (2.3)	2 (1.6)	1 (0.8)	-
**10. Do you need to force your child to eat or drink?**	**Most of the time**						**Never**
Total	3 (2.3)	4 (3.1)	-	4 (3.1)	7 (5.5)	29 (22.7)	81 (63.3)
Traditional	1 (0.8)	2 (1.6)	-	1 (0.8)	3 (2.3)	6 (4.7)	30 (23.4)
BLISS	2 (1.6)	1 (0.8)	-	3 (2.3)	2 (1.6)	12 (9.4)	25 (19.5)
Mixed	-	1 (0.8)	-	-	2 (1.6)	11 (8.6)	26 (20.3)
**11. How is your child's chewing (and sucking) ability?**	**Good**						**Very poor**
Total	91 (71.1)	24 (18.8)	5 (3.9)	1 (0.8)	1 (0.8)	3 (2.3)	3 (2.3)
Traditional	31 (24.2)	6 (4.7)	3 (2.3)	-	1 (0.8)	1 (0.8)	1 (0.8)
BLISS	30 (23.4)	10 (7.8)	1 (0.8)	1 (0.8)	-	2 (1.6)	1 (0.8)
Mixed	30 (23.4)	8 (6.3)	1 (0.8)	-	-	-	1 (0.8)
**12. What do you think about your child's growth?**	**Not growing much**						**Growing well**
Total	1 (0.8)	-	2 (1.6)	6 (4.7)	9 (7)	18 (14.1)	92 (71.9)
Traditional	-	-	1 (0.8)	1 (0.8)	4 (3.1)	6 (4.7)	31 (24.2)
BLISS	-	-	1 (0.8)	2 (1.6)	2 (1.6)	6 (4.7)	34 (26.6)
Mixed	1 (0.8)	-	-	3 (2.3)	3 (2.3)	6 (4.7)	27 (21.1)
**13. How does your child's feeding influence your relationship with them?**	**Very negatively**						**No influence at all**
Total	1 (0.8)	-	2 (1.6)	17 (13.3)	17 (13.3)	27 (21.1)	64 (50)
Traditional	1 (0.8)	-	-	6 (4.7)	10 (7.8)	6 (4.7)	20 (15.6)
BLISS	-	-	1 (0.8)	5 (3.9)	4 (3.1)	11 (8.6)	24 (18.8)
Mixed	-	-	1 (0.8)	6 (4.7)	3 (2.3)	10 (7.8)	20 (15.6)
**14. How does your child's feeding influence your family relationships?**	**No influence at all**						**Very negatively**
Total	50 (39.1)	31 (24.2)	18 (14.1)	14 (10.9)	10 (7.8)	5 (3.9)	-
Traditional	21 (16.4)	7 (5.5)	8 (6.3)	3 (2.3)	4 (3.1)	-	-
BLISS	14 (10.9)	15 (11.7)	6 (4.7)	5 (3.9)	3 (2.3)	2 (1.6)	-
Mixed	15 (11.7)	9 (7)	4 (3.1)	6 (4.7)	3 (2.3)	3 (2.3)	-

Caption: BLISS = Baby-Led Introduction to Solids; EBAI = Brazilian Child Feeding Scale

Considering aspects of general health, 43 (33.6%) children had some disease during the 12 months of life, with a higher prevalence for respiratory and allergic diseases. There was no significant difference in the EBAI score between CF methods regarding the occurrence of diseases (p > 0.05).

## DISCUSSION

The CF method was unrelated to signs and symptoms indicative of PFD in the study sample, regarding either quantitative data from the overall score for each CF introduction group or the analysis of the 14 questionnaire items. However, the children from the mixed method group, which also considers the child's autonomy, had more favorable behaviors concerning CF.

The literature has addressed the prevalence of PFD mostly in studies involving children with developmental disorders, especially considering the medical condition, to the detriment of the nutritional, dietary, and/or psychosocial condition^(8)^. Due to the cultural and social relationship linked to eating behavior and development, priority was given to listing national studies^(11,15,16)^ or those that used the same scale as the present study^(17,18)^, as well as two more recent studies that used the same scale and addressed the relationship with CF^(19,20)^.

Two Brazilian studies sought to identify feeding difficulties in neurotypical children older than 2 years, using protocols different from the one used in this research. One study investigated the prevalence in schoolchildren and found that 25.1% of the mothers participating in the study reported that their children had PFD. The authors assessed the feeding issues of the sample and concluded that 37.2% of the sample had feeding difficulties^(15)^. Another study also assessed children’s eating behaviors and concluded that 51% of those in the sample had alterations in feeding issues^(16)^.

The national study that validated, adapted, and translated the protocol that resulted in the EBAI was conducted with 242 children aged 6 months to 6 years and 11 months. The sample was divided into a control group and a case group, composed respectively of healthy children and children referred for speech-language-hearing assessment at a municipal hospital in Porto Alegre. As a result, PFD was present in 79% of the case group and 13% of the control group, both based on EBAI results^(11)^. The data from the present study corroborate the data described above, which is worthy of consideration since the sample used the same screening protocol and is from the same region, considering regional and social aspects.

Another two studies conducted in South Africa sought to identify PFD in children using the same protocol as this study. One study aimed to determine the relationship between feeding and development in infants with an average age of 8.8 months. Based on MCH-FS results, the authors concluded that 4.9% of the sample had feeding difficulties, divided between mild (71.4%) and moderate to severe (28.6%). In the qualitative analysis, caregivers considered that their children were growing well and that feeding did not influence their relationship with family members^(17)^. The other study aimed to describe feeding characteristics and determine the nature of feeding difficulties in 200 children aged 6 to 12 months. As a result, data showed that 6.5% of the children had feeding difficulties (61.5% mild, 15.4% moderate, and 23.1% severe). Finally, regarding the growth of their children, the caregivers reported that they were growing well, and the percentage of most questions came close to the maximum limit^(18)^. The percentage of children who presented PFD in this study was higher than in both aforementioned studies, which can be explained by regional and socioeconomic differences. However, data in the qualitative analysis of the questions in the present study corroborate the studies above.

Recently, two Polish studies by the same team^(19,20)^ used the MCH-FS as an instrument to identify PFD (described in the study as food neophobia) in children who were subjected to two CF groups, named BLW and non-BLW. The two studies encompassed children aged 2 to 7 years; the first aimed to assess the prevalence of PFD, and the second aimed to verify the influence of the CF method on PFD, an objective similar to the present study.

Children who received food using the traditional CF method were at higher risk for adverse eating issues, while those with BLW were not. The authors did not notice differences regarding appetite and the variety of foods. However, caregivers in the BLW group did not need to stimulate the children to eat, differing from the non-BLW group. Moreover, the latter showed a propensity to choose food according to their mothers' preferences^(19,20)^.

The Polish studies claim that opting for the complete BLW method during CF can protect the child against future feeding problems, such as food selectivity or selective eating. The present research chose the BLISS method over BLW due to the advantages described in the literature^(7)^.

In the present study, the mixed method, which gives the child total autonomy in the choice, presented the least prevalence of feeding difficulties. The BLISS method points to the autonomy of children regarding how much and when to eat, but not how it will be offered. This study considered it fundamental to approach the children’s individual characteristics more broadly, also offering them the choice of how the food is presented. Their individual characteristics are respected as the main point in CF^(5)^. The literature frequently associates the attitude of parents and/or caregivers in CF with children’s food rejection^(21)^.

It is also worth noting that passive food offering can limit a child's development, impair the development of chewing and swallowing skills, and increase the risk of food selectivity. The lack of progression in food consistency prevents the child from reaching their full potential for developing oral motor and fine motor skills, which are offered by alternative methods. Exploring different textures, expanding sensory aspects, and handling food orally are fundamental to development^(22)^. The present research made sure that the groups were homogeneous in previous and current aspects regarding nutritive and non-nutritive sucking habits, as these can interfere with oral motor skills.

It should be noted that the study sample consisted of mothers of children who received guidance on feeding, had high education levels and family income above the national average, and were constantly present during their children's mealtimes, as these factors may interfere with the results. Another limitation is the fact that the follow-up questionnaires were applied remotely and self-answered.

Finally, further studies are needed to verify the relationship between CF methods and PFD in different populations.

## CONCLUSION

In the study sample, 12.5% of the children had PFD and favorable performance in the 14 questions on the behavior of the children and caregivers in relation to feeding aspects. CF methods were not associated with PFD, but the disorder was less prevalent in the mixed method.
