# Supplementary material for: Complementary feeding introduction methods and pediatric feeding disorder in the first year of life: a randomized clinical trial
Source: Codas. 2026 Jul 16;38(5):e20250265. doi: 10.1590/2317-1782/e20250265en (PMC13440684; doi:10.1590/2317-1782/e20250265en)
Supplement: Translation

# Translation: Métodos de introdução da alimentação complementar e distúrbio alimentar pediátrico no primeiro ano de vida: um ensaio clínico randomizado

**DOI:** 10.1590/2317-1782/e20250265pt

**Authors:** Letícia Cristine Welke, Adriela Azevedo Souza Mariath, Juliana Rombaldi Bernardi, Leandro Meirelles Nunes, Erissandra Gomes

**Affiliations:** 1 Curso de Graduação em Fonoaudiologia, Faculdade de Odontologia, Universidade Federal do Rio Grande do Sul – UFRGS - Porto Alegre (RS), Brasil.; 2 Departamento de Cirurgia e Ortopedia, Faculdade de Odontologia, Universidade Federal do Rio Grande do Sul – UFRGS - Porto Alegre (RS), Brasil.; 3 Departamento de Nutrição, Faculdade de Medicina, Universidade Federal do Rio Grande do Sul – UFRGS - Porto Alegre (RS), Brasil.; 4 Hospital de Clínicas de Porto Alegre - Porto Alegre (RS), Brasil.; 5 Departamento de Pediatria, Faculdade de Medicina, Universidade Federal do Rio Grande do Sul – UFRGS - Porto Alegre (RS), Brasil.

**Keywords:** Comportamento Alimentar, Lactente, Alimentação Infantil, Alimentação Complementar, Métodos de Alimentação

## Abstract

**Objetivo:**

Verificar se há relação entre o método de introdução da alimentação complementar (AC) e o distúrbio alimentar pediátrico (DAP), no primeiro ano de vida.

**Método:**

Ensaio clínico com 128 pares mãe-crianças randomizados em grupos de intervenção por métodos de AC: tradicional; Baby-Led Introduction to Solids (BLISS); ou misto. A intervenção foi realizada aos cinco meses e meio de idade do lactente, em cozinha experimental com as refeições preparadas segundo o método randomizado. Houve seguimento aos sete e nove meses de idade dos lactentes. Aos 12 meses foi aplicada a Escala Brasileira de Alimentação Infantil (EBAI) para rastreio do DAP. Este estudo foi registrado no Brazilian Clinical Trials Registry (ReBEC).

**Resultados:**

O DAP esteve presente em 16 (12,5%) crianças: 5 (3,9%) do método tradicional (3 com DAP de grau leve e 2 de grau severo), 9 (7%) do método BLISS (5 com DAP de grau leve, 2 de grau moderado e 2 de grau severo) e 2 (1,6%) do método misto (ambos de grau leve). Na análise das 14 questões que compõem o EBAI, as respostas tenderam para o aspecto adequado e/ou favorável para todos os métodos de IAC, sem diferenças estatisticamente significativas.

**Conclusão:**

O DAP não foi influenciado pelo método de AC na amostra pesquisada.

## INTRODUÇÃO

A introdução da alimentação complementar (AC) ao leite materno ou fórmula infantil é realizada com a apresentação de outros líquidos e, especialmente, de alimentos sólidos. Para que a AC ocorra, os lactentes devem apresentar sinais de prontidão e habilidades associadas ao controle cervical e motor global amplo e fino, evolução dos aspectos orais, demonstração de interesse pelos alimentos, além de maturação gastrointestinal e do sistema imunológico^(1,2)^.

O Ministério da Saúde recomenda que a AC seja iniciada aos seis meses de idade do lactente, com o controle quanto à ingestão da criança realizada pelo cuidador, ofertando alimentos inicialmente na textura de papas e/ou purês, com uso de utensílios, aproximando-se gradualmente à consistência alimentar da família com um ano de idade^(1)^. Esse método tem sido descrito como tradicional ou *Parent-Led Weaning* (*PLW*), entretanto nos últimos anos a literatura tem considerado outros métodos de AC que promovem a autonomia da criança^(3-5)^. O *Baby-Led Weaning* (*BLW*) é um método de introdução da AC que visa permitir que a criança guie o momento de alimentação, com objetivo principal de desenvolver a noção de saciedade e autonomia, sendo os alimentos ofertados em formato de tiras ou bastões e sem adaptações de suas consistências^(6)^. Considerando os questionamentos apontados por pesquisadores e profissionais de saúde em relação ao método BLW, foi criada uma versão modificada, denominada de *Baby-Led Introduction to SolidS (BLISS)*. As recomendações BLISS são as mesmas do método BLW, contudo, com alguns aspectos adicionais: garantir uma fonte de alimentos ricos em ferro em cada refeição; oferecer um alimento de alto teor calórico em cada refeição; oferecer alimentos preparados em uma textura e formato que reduz o risco de engasgo e evitar alimentos listados como de alto risco de aspiração^(7)^.

No decorrer do processo de AC do lactente podem ocorrer dificuldades alimentares que, se não observadas e orientadas de maneira adequada, podem resultar no Distúrbio Alimentar Pediátrico (DAP) e se perpetuar ao longo do desenvolvimento infantil. O DAP é definido como ingestão oral prejudicada ou não apropriada para a idade, associada a condição médica, nutricional, alimentar e/ou psicossocial^(8)^. O DAP pode ocorrer tanto em crianças com desenvolvimento neurotípico, com prevalência de 25-35%, como naquelas com comorbidades, em até 80%. As manifestações clínicas vão desde comportamentos atípicos até aspectos clínicos alterados, afetando o desenvolvimento global e repercutindo nas questões biopsicossociais, especialmente nas relações familiares e na qualidade de vida^(9,10)^. Apesar de haver diferentes protocolos para triagem de DAP na literatura, um dos mais utilizados e o único validado no Brasil é a *Montreal Children’s Hospital Feeding Scale* (*MCH-FS*), denominada nacionalmente como Escala Brasileira de Alimentação Infantil (EBAI)^(11)^.

A AC é motivo de grande preocupação para os pais e/ou responsáveis dos lactentes, principalmente quando há dificuldades alimentares que ocorrem no processo de introdução alimentar, que podem culminar em DAP e ocasionar diversas consequências à criança^(12)^. Considerando que a AC é um momento importante no desenvolvimento infantil e para um adequado relacionamento da criança com a alimentação, este estudo justifica-se pela importância de pesquisas voltadas à relação entre os métodos de AC e o DAP. Assim, o objetivo deste estudo foi verificar se há relação entre o método de introdução da AC e o DAP, no primeiro ano de vida do lactente.

## MÉTODOS

Esta pesquisa faz parte de um estudo maior que foi aprovado no Comitê de Ética em Pesquisa do Hospital de Clínicas de Porto Alegre sob o parecer nº 3.094.555 (CAAE: 01537018.5.0000.5327) e registrado no *Brazilian Clinical Trials Registry (*ReBEC) com número RBR-229SCM. As mães das crianças concordaram em participar do estudo, por meio do Termo de Consentimento Livre Esclarecido. O protocolo do estudo ao qual este pertence foi previamente publicado^(5)^. Trata-se de um ensaio clínico randomizado controlado, com três braços de intervenção em relação ao método de introdução da AC:

- método tradicional^(1)^: pais foram orientados a iniciar a oferta da alimentação complementar por colher pelo adulto a partir do sexto mês de vida, de forma lenta e gradual. Oferecer alimentos complementares (cereais, tubérculos, carnes, leguminosas, frutas e verduras) três vezes ao dia, sem horários rígidos e respeitando o apetite, aumentando a oferta ao longo dos meses; a consistência da alimentação deve ser inicialmente pastosa, amassada com garfo (de seis a oito meses na forma de mingau e purê) e progredir gradativamente até atingir a consistência da alimentação da família aos 12 meses de idade, com variedade de cores e grupos de alimentos em todas as refeições, sem misturar ou peneirar os alimentos;
- método *BLISS* ^(5)^: pais foram orientados a incentivar o lactente a se alimentar sozinho, mas sempre acompanhado por um adulto e participando das refeições em família. A consistência da alimentação oferecida desde os seis meses de vida deve ser firme, em formas que permitam que o lactente se alimente com as próprias mãos; cortados em formas alongadas, como tiras ou palitos, para facilitar o movimento de pinça dos dedos e evitar engasgos. Foi aconselhado respeitar o tempo da criança para explorar os sabores e texturas, e oferecer três tipos de alimentos em cada refeição: um alimento fonte de ferro (por exemplo, carne vermelha), um alimento fonte de energia e uma fonte alimentar de fibra, como frutas ou vegetais;
- método misto^(1)^: pais foram orientados a aplicar inicialmente o método *BLISS*. Caso a criança não estivesse satisfeita ou desinteressada pelo alimento, foi orientado oferecer o alimento pelo método tradicional durante a mesma refeição. Os cuidadores foram treinados e receberam material impresso sobre como perceber os sinais de satisfação do filho. Trata-se de abordagem que considera o desejo e o comportamento da criança.

A amostra foi composta por mães residentes em Porto Alegre e região metropolitana, e por seus filhos saudáveis, nascidos a termo, não gemelares, com peso ao nascer maior ou igual a 2.500 gramas e que não tivessem iniciado a AC. Foram considerados critérios de exclusão crianças que apresentassem malformações congênitas, déficits neurológicos ou restrições alimentares no primeiro ano de vida. O recrutamento ocorreu por meio de convite *online* nas redes sociais, páginas e grupos relacionados a mães, bem como por cartazes de divulgação em locais pertinentes ao tema da pesquisa. A randomização da amostra ocorreu em blocos de três, até que fosse atingido o número de pares calculado, utilizando o site Randomization^(13)^ por um pesquisador não envolvido nas coletas e análise.

A partir da randomização dos grupos, foram realizados encontros para a intervenção com grupos de quatro a oito mães quando os lactentes completassem cinco meses e meio de idade, em uma clínica de nutrição, com uma nutricionista e uma fonoaudióloga. Em virtude da pandemia pelo Covid-19, algumas intervenções ocorreram por via remota, por vídeo-chamada em aplicativos de comunicação virtual com as mesmas orientações e equipe envolvidas na intervenção presencial. Houve reforço do aconselhamento em alimentação infantil, realizado por meio de contato telefônico aos sete meses e por visitas domiciliares aos nove meses, e aos 12 meses de vida foram realizadas avaliações, em um encontro presencial ou por via remota. Para este estudo foram utilizados os dados coletados no questionário inicial para a caracterização da amostra e os dados coletados aos 12 meses para obtenção de dados de saúde geral, alimentares e orais, assim como a aplicação da EBAI. Ambos foram aplicados de forma remota e auto respondidos pelos pais individualmente; após enviado para os pesquisadores, que estavam à disposição caso houvesse alguma dúvida no preenchimento.

O EBAI é um instrumento que objetiva avaliar o comportamento alimentar de crianças. O protocolo foi publicado no Canadá, em 2011, com o nome de *Montreal Children’s Hospital Feeding Scale* (*MCH-FS*). A *MCH-FS* classifica a dificuldade a partir dos escores obtidos, ou seja, *T-escore* entre 61 e 65 é classificada como dificuldade leve, 66 a 70 a dificuldade é moderada e acima de 70 a dificuldade é severa. Caracteriza-se por ser uma ferramenta de triagem simples e rápida de problemas alimentares de crianças de seis meses a seis anos e 11 meses de idade. Possui 14 itens a serem respondidos pelo responsável que esteja presente na maior parte das refeições da criança, com respostas que variam de 1 a 7, mas que cada variável tem uma classificação. Os itens são relacionados aos sintomas sensório-motores orais, sensoriais orais, apetite, preocupações e comportamentos no momento da refeição, estratégias utilizadas e reações dos cuidadores^(14)^. Desde 2011, a *MCH-FS* foi validada no Canadá, seu país de origem, e em mais outros países; no Brasil foi validada e adaptada para o português brasileiro, resultando na EBAI^(11)^.

O banco de dados foi elaborado utilizando o *Statistical Package for the Social Sciences* (*SPSS*) versão 21.0, com entrada dupla e posterior validação. Para manter o anonimato, cada par de mãe e bebê foi identificado com um número. As variáveis categóricas foram apresentadas através de percentual relativo e absoluto e as variáveis quantitativas através de média (desvio padrão), mediana (mínima e máxima) ou mediana (P_25_ e P_75_). Foi utilizado o valor de significância de p de 5%, adotando o teste do Qui-quadrado de Pearson e teste ANOVA.

## RESULTADOS

A amostra foi composta por 128 díades mães-lactentes, divididas pelo método de AC: 43 (33,6%) no método tradicional, 45 (35,1%) no método *BLISS* e 40 (31,2%) no método misto, conforme o fluxograma apresentado na Figura 1. Os dados de caracterização inicial da amostra total e pelo método de AC estão descritos na Tabela 1; e na Tabela 2 estão os dados aos 12 meses. Comparando as variáveis descritas com os métodos de AC não houve diferença significativa, exceto para a variável “frequenta a creche" (p< 0,05), mas sem repercussão na análise final comparativa.

**Figura 1:** Fluxograma do estudo

Legenda: BLISS = Baby-Led Introduction to Solids

**Tabela 1:** Caracterização da amostra total e por método de introdução da AC, aos 5 meses e meio de idade (n=128)

**Variáveis**	**Total**	**Métodos**			**Valor p**
		**Tradicional n=43**	**BLISS n=45**	**Misto n=40**	
**Sexo da criança** – n(%)					
Masculino	58(45,3)	17(39,5)	21(46,7)	20(50)	0,617^*^
Feminino	70(54,7)	26(60,5)	24(53,3)	20(50)	
**Escolaridade materna** – média±desvio-padrão	17,6±5,3	16,9±5,3	18±6,1	17,9±4,4	0,627**
**Raça materna** – n(%)					
Branca	109(85,2)	35(81,4)	38(84,4)	36(90)	0,697
Outras	19(14,8)	8(18,6)	7(15,6)	4(10)	
**Renda familiar (R$)** – Mediana	6000	5000	8000	6000	0,164^**^
(P25-P75)	(4000 - 10000)	(3700 - 10000)	(4000 - 14500)	(4550 - 10000)	

* Teste do Qui-quadrado de Pearson;

** Teste ANOVA; p<0,05

Legenda: BLISS = *Baby-Led Introduction to SolidS*; AC = alimentação complementar

**Tabela 2:** Caracterização da amostra total e por método de introdução da AC, aos 12 meses (n=128)

**Variáveis**	**Total**	**Métodos**			
		**Tradicional n=43**	**BLISS n=45**	**Misto n=40**	**Valor p**
**Peso da criança (kg)** – média±desvio-padrão	9,6±1,2	9,8±1,4	9,5±1,0	9,6±1,1	0,582**
**Idade da introdução AC (em dias)** – média±desvio-padrão	172,5±24,2	168,2±28,7	174,0±22,7	175,5±20,1	0,343^**^
**Frequenta creche** – n(%)	37(28,9)	18(41,9)	12(26,7)	7(17,5)	0,046^*^
**Está em AM –** n(%)	96(75)	34(79)	34(75,5)	28(70)	0,631*
**Faz uso de mamadeira** – n(%)	77(60,2)	27(62,8)	30(66,7)	20(50)	0,267*
**Faz uso de chupeta** – n(%)	42(32,8)	14(32,5)	18(40)	10(25)	0,339*

* Teste do Qui-quadrado de Pearson;

** Teste ANOVA; p<0,05

Legenda: BLISS = *Baby-Led Introduction to Solids*; AM = aleitamento materno; AC = alimentação complementar

A pontuação do questionário EBAI está descrita na Tabela 3. Não houve diferença estatisticamente significativa (p>0,05) entre os diferentes métodos de AC. Considerando o ponto de corte do *T-score* para DAP, verificou-se que 16 (12,5%) crianças apresentaram DAP: 5 (3,9%) eram pertencentes ao método tradicional (3 com DAP de grau leve e 2 de grau severo), 9 (7%) ao método BLISS (5 com DAP de grau leve, 2 de grau moderado e 2 de grau severo) e 2 (1,6%) ao método misto (ambas classificadas como de grau leve).

**Tabela 3:** EBAI, por amostra total e por método de introdução AC aos 12 meses de idade (n=128)

**Variáveis**	**Total**	**Métodos**			
		**Tradicional n=43**	**BLISS n=45**	**Misto n=40**	**Valor p** ^*^
Pontuação					0,394
média±desvio padrão	51,4±7,6	51,2±8,3	52,5±8,5	50,3±5,5	
IC 95%	50,1-52,7	48,6-53,7	50-55,1	48,6-52,1	
mediana (mínima - máxima)	50 (36-74)	50 (36-74)	51 (40-72)	49 (39-64)	

* Teste ANOVA; p<0,05

Legenda: BLISS = *Baby-Led Introduction to Solids*; EBAI = Escala Brasileira de Alimentação Infantil; IC = intervalo de confiança; AC = alimentação complementar

No Quadro 1 estão descritas as 14 variáveis, considerando as respostas para cada uma das questões. Na análise das respostas, percebe-se que a maioria da amostra, independente do grupo que pertence, atribui características favoráveis ao processo de AC. Não foram encontradas diferenças significativas (p>0,05) entre os métodos de AC para as questões.

**Quadro 1:** Distribuição absoluta e relativa das respostas ao EBAI (n=128)

**Variáveis**	**Respostas**						
	**1**	**2**	**3**	**4**	**5**	**6**	**7**
	**n (%)**	**n (%)**	**n (%)**	**n (%)**	**n (%)**	**n (%)**	**n (%)**
**1. O que acha dos momentos de refeições com a criança?**	**Muito difícil**						**Fácil**
Total	-	2 (1,6)	13 (10,2)	22 (17,2)	27 (21,1)	34 (26,6)	30 (23,4)
Tradicional	-	2 (1,6)	1 (0,8)	8 (6,3)	11 (8,6)	11 (8,6)	10 (7,8)
BLISS	-	-	10 (7,8)	10 (7,8)	6 (4,7)	8 (6,3)	11 (8,6)
Misto	-	-	2 (1,6)	4 (3,1)	10 (7,8)	15 (11,7)	9 (7)
**2. Quão preocupado você está com a alimentação da sua criança?**	**Não estou preocupado**						**Estou muito preocupado**
Total	10 (7,8)	10 (7,8)	9 (7)	14 (10,9)	26 (20,3)	23 (18)	36 (28,1)
Tradicional	4 (3,1)	3 (2,3)	2 (1,6)	7 (5,5)	8 (6,3)	6 (4,7)	13 (10,2)
BLISS	3 (2,3)	4 (3,1)	4 (3,1)	3 (2,3)	8 (6,3)	9 (7)	14 (10,9)
Misto	3 (2,3)	3 (2,3)	3 (2,3)	4 (3,1)	10 (7,8)	8 (6,3)	9 (7)
**3. Quanto de apetite (fome) sua criança tem?**	**Nunca tem fome**						**Tem um bom apetite**
Total	-	4 (3,1)	6 (4,7)	17 (13,3)	24 (18,8)	29 (22,7)	48 (37,5)
Tradicional	-	2 (1,6)	3 (2,3)	3 (2,3)	6 (4,7)	10 (7,8)	19 (14,8)
BLISS	-	1 (0,8)	2 (1,6)	9 (7)	9 (7)	7 (5,5)	17 (13,3)
Misto	-	1 (0,8)	1 (0,8)	5 (3,9)	9 (7)	12 (9,4)	12 (9,4)
**4. Quando a sua criança começa a se recusar a comer durante as refeições?**	**No início da refeição**						**No fim da refeição**
Total	6 (4,7)	5 (3,9)	7 (5,5)	19 (14,8)	20 (15,6)	29 (22,7)	42 (32,8)
Tradicional	3 (2,3)	2 (1,6)	1 (0,8)	7 (5,5)	4 (3,1)	10 (7,8)	16 (12,5)
BLISS	1 (0,8)	2 (1,6)	4 (3,1)	10 (7,8)	8 (6,3)	7 (5,5)	13 (10,2)
Misto	2 (1,6)	1 (0,8)	2 (1,6)	2 (1,6)	8 (6,3)	12 (9,4)	13 (10,2)
**5. Quanto tempo (em minutos) dura a refeição da sua criança?**	**01-10**	**11-20**	**21-30**	**31-40**	**41-50**	**51-60**	**> 60**
Total	17 (13,3)	43 (33,6)	42 (32,8)	16 (12,5)	6 (4,7)	2 (1,6)	2 (1,6)
Tradicional	12 (9,4)	11 (8,6)	9 (7)	7 (5,5)	2 (1,6)	1 (0,8)	1 (0,8)
BLISS	2 (1,6)	16 (12,5)	19 (14,8)	4 (3,1)	3 (2,3)	1 (0,8)	-
Misto	3 (2,3)	16 (12,5)	14 (10,9)	5 (3,9)	1 (0,8)	-	1 (0,8)
**6. Como sua criança se comporta durante a refeição?**	**Se comporta muito bem**						**Faz grande bagunça, birra, manha**
Total	21 (16,4)	35 (27,3)	19 (14,8)	25 (19,5)	18 (14,1)	5 (3,9)	5 (3,9)
Tradicional	10 (7,8)	8 (6,3)	5 (3,9)	11 (8,6)	5 (3,9)	3 (2,3)	1 (0,8)
BLISS	5 (3,9)	13 (10,2)	8 (6,3)	7 (5,5)	7 (5,5)	1 (0,8)	4 (3,1)
Misto	6 (4,7)	14 (10,9)	6 (4,7)	7 (5,5)	6 (4,7)	1 (0,8)	-
**7. A sua criança nauseia, cospe ou vomita com algum tipo de alimento**	**Nunca**						**Na maioria das vezes**
Total	41 (32)	53 (41,4)	13 (10,2)	9 (7)	8 (6,3)	2 (1,6)	2 (1,6)
Tradicional	12 (9,4)	18 (14,1)	5 (3,9)	3 (2,3)	2 (1,6)	2 (1,6)	1 (0,8)
BLISS	11 (8,6)	20 (15,6)	6 (4,7)	3 (2,3)	4 (3,1)	-	1 (0,8)
Misto	18 (14,1)	15 (11,7)	2 (1,6)	3 (2,3)	2 (1,6)	-	-
**8. A sua criança fica com a comida parada na boca?**	**Na maioria das vezes**						**Nunca**
Total	2 (1,6)	1 (0,8)	10 (7,8)	5 (3,9)	10 (7,8)	39 (30,5)	61 (47,7)
Tradicional	-	-	4 (3,1)	2 (1,6)	5 (3,9)	13 (10,2)	19 (14,8)
BLISS	1 (0,8)	1 (0,8)	2 (1,6)	3 (2,3)	3 (2,3)	12 (9,4)	23 (18)
Misto	1 (0,8)	-	4 (3,1)	-	2 (1,6)	14 (10,9)	19 (14,8)
**9. Você precisa ir atrás da sua criança ou usar distrações (como por exemplo brinquedos, TV) durante a refeição para que ele coma?**	**Nunca**						**Na maioria das vezes**
Total		26 (20,3)	15 (11,7)	6 (4,7)	10 (7,8)	6 (4,7)	
Tradicional		6 (4,7)	4 (3,1)	1 (0,8)	5 (3,9)	1 (0,8)	3 (2,3)
BLISS	62 (48,4)	10 (7,8)	4 (3,1)	2 (1,6)	3 (2,3)	4 (3,1)	2 (1,6)
Misto	24 (18,8)	10 (7,8)	7 (5,5)	3 (2,3)	2 (1,6)	1 (0,8)	1 (0,8)
	21 (16,4)						-
	17 (13,3)						
**10. Você precisa forçar sua criança a comer ou beber?**	**Na maioria das vezes**						**Nunca**
Total	3 (2,3)	4 (3,1)	-	4 (3,1)	7 (5,5)	29 (22,7)	81 (63,3)
Tradicional	1 (0,8)	2 (1,6)	-	1 (0,8)	3 (2,3)	6 (4,7)	30 (23,4)
BLISS	2 (1,6)	1 (0,8)	-	3 (2,3)	2 (1,6)	12 (9,4)	25 (19,5)
Misto	-	1 (0,8)	-	-	2 (1,6)	11 (8,6)	26 (20,3)
**11. Como é a habilidade de mastigação (e sucção) da sua criança?**	**Boa**						**Muito Ruim**
Total	91 (71,1)	24 (18,8)	5 (3,9)	1 (0,8)	1 (0,8)	3 (2,3)	3 (2,3)
Tradicional	31 (24,2)	6 (4,7)	3 (2,3)	-	1 (0,8)	1 (0,8)	1 (0,8)
BLISS	30 (23,4)	10 (7,8)	1 (0,8)	1 (0,8)	-	2 (1,6)	1 (0,8)
Misto	30 (23,4)	8 (6,3)	1 (0,8)	-	-	-	1 (0,8)
**12. O que você acha do crescimento da sua criança?**	**Crescendo pouco**						**Crescendo bem**
Total	1 (0,8)	-	2 (1,6)	6 (4,7)	9 (7)	18 (14,1)	92 (71,9)
Tradicional	-	-	1 (0,8)	1 (0,8)	4 (3,1)	6 (4,7)	31 (24,2)
BLISS	-	-	1 (0,8)	2 (1,6)	2 (1,6)	6 (4,7)	34 (26,6)
Misto	1 (0,8)	-	-	3 (2,3)	3 (2,3)	6 (4,7)	27 (21,1)
**13. Como a alimentação da sua criança influencia sua relação com ela?**	**De forma muito negativa**						**Não influencia nada**
Total	1 (0,8)	-	2 (1,6)	17 (13,3)	17 (13,3)	27 (21,1)	64 (50)
Tradicional	1 (0,8)	-	-	6 (4,7)	10 (7,8)	6 (4,7)	20 (15,6)
BLISS	-	-	1 (0,8)	5 (3,9)	4 (3,1)	11 (8,6)	24 (18,8)
Misto	-	-	1 (0,8)	6 (4,7)	3 (2,3)	10 (7,8)	20 (15,6)
**14. Como a alimentação da sua criança influencia as suas relações familiares?**							**De forma muito negativa**
Total	50 (39,1)	31 (24,2)	18 (14,1)	14 (10,9)	10 (7,8)	5 (3,9)	-
Tradicional	21 (16,4)	7 (5,5)	8 (6,3)	3 (2,3)	4 (3,1)	-	-
BLISS	14 (10,9)	15 (11,7)	6 (4,7)	5 (3,9)	3 (2,3)	2 (1,6)	-
Misto	15 (11,7)	9 (7)	4 (3,1)	6 (4,7)	3 (2,3)	3 (2,3)	

Legenda: BLISS = *Baby-Led Introduction to Solids*; EBAI = Escala Brasileira de Alimentação Infantil

Considerando os aspectos de saúde geral, 43 (33,6%) das crianças apresentaram alguma doença ao longo dos 12 meses de vida, com maior prevalência para as doenças respiratórias e alérgicas. Não houve diferença significativa na pontuação do EBAI comparando o método de AC, com o fato de ter apresentado doença (p>0,05).

## DISCUSSÃO

Na amostra pesquisada, tanto para os dados apresentados quantitativamente através da pontuação geral para cada grupo de introdução da AC, como da análise das 14 alternativas do questionário, verificou-se que o método de AC não se relacionou com o fato de apresentar ou não sinais e sintomas indicativos de DAP. No entanto, as crianças que estavam no grupo do método misto, que também considera a autonomia da criança, tiveram comportamentos mais favoráveis em relação à AC.

A literatura tem abordado a prevalência de DAP, em sua maioria, em estudos envolvendo crianças com alterações no desenvolvimento, considerando especialmente a condição médica, em detrimento à nutricional, alimentar e/ou psicossocial^(8)^. Em função da relação cultural e social atreladas ao comportamento e desenvolvimento alimentar, priorizou-se em elencar estudos nacionais^(11,15,16)^ ou que utilizaram a mesma escala do presente estudo^(17,18)^. Além desses, dois estudos mais atuais que, além de utilizarem a mesma escala, abordaram a relação com a AC^(19,20)^.

Dois estudos brasileiros buscaram identificar as dificuldades alimentares em crianças neurotípicas maiores de dois anos de idade, utilizando protocolos diferentes do utilizado neste estudo. Um estudo pesquisou a prevalência em crianças com idade escolar e obteve que 25,1% das mães participantes do estudo referiram que seus filhos possuíam DAP. Os autores realizaram avaliação das questões alimentares da amostra e com isso puderam concluir que 37,2% da amostra apresentava dificuldade alimentar^(15)^. Outro estudo também avaliou os comportamentos alimentares de crianças e concluiu que 51% das crianças da amostra possuíam alterações nas questões alimentares^(16)^.

Outro estudo nacional é o de validação, adaptação e tradução do protocolo utilizado, que resultou no EBAI. O estudo foi realizado com 242 crianças com idades entre seis meses e seis anos e 11 meses. A amostra foi dividida em grupo controle e grupo de casos, compostos respectivamente por crianças saudáveis e crianças encaminhadas para avaliação fonoaudiológica em um hospital municipal da cidade de Porto Alegre. Como resultados, 79% do grupo de casos apresentaram DAP e 13% do grupo controle, ambos a partir dos resultados obtidos com a aplicação da EBAI^(11)^. Os dados do presente estudo corroboram com os dados descritos acima, fato a ser considerado, pois a amostra utilizou o mesmo protocolo para triagem e é da mesma região, o que considera aspectos regionais e sociais.

Outros dois estudos realizados na África do Sul buscaram identificar DAP em crianças através do mesmo protocolo deste estudo. Um estudo buscou determinar a relação entre a alimentação e desenvolvimento de bebês com idade média de 8,8 meses de idade. A partir dos resultados da *MCH-FS* os autores concluíram que 4,9% da amostra possuía dificuldade alimentar, divididos entre grau leve (71,4%) e grau moderado a severo (28,6%). Na análise qualitativa, os responsáveis consideraram que suas crianças estavam crescendo bem e que a alimentação não influenciava em sua relação ou com os familiares^(17)^. O outro estudo buscou descrever características alimentares e determinar a natureza das dificuldades alimentares de 200 crianças com idades entre seis e 12 meses. Como resultados, obtiveram-se dados de que 6,5% das crianças apresentaram dificuldade alimentar (61,5% de grau leve, 15,4% de grau moderado e 23,1% de grau severo). Por fim, em relação ao crescimento de suas crianças, os responsáveis referiram que elas estavam crescendo bem e o percentual da maioria das questões chegou próximo ao limite máximo^(18)^. O percentual de crianças que apresentaram DAP neste estudo foi maior que em ambos os estudos supracitados, o que pode ser explicado pela diferença regional e socioeconômica. Entretanto, na análise qualitativa das questões, os dados do presente estudo corroboram com os estudos acima.

Recentemente, dois estudos poloneses da mesma equipe^(19,20)^ utilizaram a MCH-FS como instrumento para a identificação de DAP (descrito no estudo como neofobia alimentar) em crianças que foram submetidas a dois grupos de AC, nomeados como *BLW* e não-*BLW*. Os dois estudos são com crianças de dois a sete anos, sendo o primeiro com o objetivo de avaliar a prevalência de DAP e o segundo com o objetivo de verificar a influência do método de AC no DAP, objetivo semelhante ao presente estudo.

Crianças que durante a AC receberam alimentos com método tradicional apresentaram maiores riscos para questões alimentares adversas, enquanto àquelas com *BLW* não. Os autores não perceberam diferenças quanto ao apetite e variedade de alimentos, porém no grupo com a AC realizada por meio do *BLW*, os responsáveis não precisavam estimular a criança a comer, diferindo do grupo não-*BLW*. Além disso, crianças do grupo não-*BLW* apresentaram propensão a escolher alimentos de acordo com a preferência das mães^(19,20)^.

Os estudos poloneses afirmam que a opção pelo método *BLW* completo durante a AC pode proteger a criança contra a ocorrência de problemas de alimentação, como seletividade alimentar ou alimentação seletiva no futuro. Na presente pesquisa optou-se pelo método *BLISS* em detrimento ao *BLW* pelas vantagens descritas na literatura^(7)^.

No presente estudo, o método considerado misto, que atribui a criança total autonomia na escolha, foi o que menos apresentou prevalência de dificuldade alimentar. O método BLISS aponta a autonomia das crianças, do ponto de vista do quanto e quando comer, mas não da forma como será ofertado. Este estudo considerou como ponto fundamental a característica individual da criança de forma mais ampla, oferecendo também à criança a escolha da forma de apresentação dos alimentos. Respeita-se a característica individual da criança como ponto principal na AC^(5)^. Na literatura a postura dos pais e/ou cuidadores na AC é um fator que frequentemente está associado à rejeição de alimentos pelas crianças^(21)^.

Ressalta-se, também, que a oferta passiva de alimentos pode limitar o desenvolvimento da criança, além de prejudicar o desenvolvimento da habilidade na mastigação e deglutição e aumentar o risco de seletividade alimentar. A falta de evolução da consistência impede que a criança atinja seu pleno potencial de desenvolvimento das habilidades motoras orais e de motricidade fina, habilidades oferecidas pelos métodos alternativos. A exploração de diferentes texturas, a ampliação dos aspectos sensoriais e a habilidade do manejo oral dos alimentos é fundamental no desenvolvimento^(22)^. Na presente pesquisa teve-se o cuidado de verificar que os grupos eram homogêneos em relação aos aspectos prévios e atuais em relação aos hábitos de sucção nutritiva e não-nutritiva, pois os mesmos podem interferir na habilidade motora oral.

Deve-se destacar que a amostra deste estudo foi composta por mães de crianças que receberam orientações sobre alimentação, possuíam alta escolaridade e renda familiar superiores à média nacional e que as mães estavam constantemente presentes nos momentos de refeições dos filhos; fatores, esses, que podem interferir nos resultados. Outra limitação é o fato dos questionários de acompanhamento terem sido aplicados remotamente e auto respondidos.

Por fim, faz-se necessária a realização de outros estudos em diferentes populações que se proponham a relacionar métodos de AC e DAP.

## CONCLUSÃO

Na amostra deste estudo, 12,5% das crianças apresentaram DAP e desempenho favorável nas 14 questões quanto ao comportamento das crianças e dos responsáveis em relação aos aspectos alimentares. Não houve associação entre os métodos de AC e o DAP, mas no método misto o distúrbio foi menos prevalente.
